# Draft genome sequence of *Bacillus thuringiensis* strain V-AB8.18, a novel isolate with potential nematicidal activity

**DOI:** 10.1128/mra.00227-24

**Published:** 2024-06-07

**Authors:** Leopoldo Palma, Yolanda Bel, Baltasar Escriche

**Affiliations:** 1 Laboratorio de Control Biotecnológico de Plagas, Departamento de Genética, Instituto BIOTECMED, Universitat de València, Burjassot-València, Spain; 2 Instituto Multidisciplinario de Investigación y Transferencia Agroalimentaria y Biotecnológica (IMITAB), Consejo Nacional de Investigaciones Científicas y Técnicas (CONICET), Universidad Nacional de Villa María (UNVM), Villa María, Argentina; University of Maryland School of Medicine, Baltimore, Maryland, USA

**Keywords:** App6, Cry5B, *Meloidogyne*, nematode, nematocidal

## Abstract

We report the draft genome of *Bacillus thuringiensis* strain V-AB8.18, comprising 308 contigs totaling 6,182,614 bp, with 35% G + C content. It contains 6,151 putative protein-coding genes, including App6 and Cry5-like crystal proteins, exhibiting 99% pairwise identity to nematicidal proteins App6Aa2 and Cry5Ba2, active against *Meloidogyne incognita* and *Meloidogyne hapla*.

## ANNOUNCEMENT

*Bacillus thuringiensis* (Bt) is a Gram-positive bacterium able to produce a variety of proteins showing biocidal activity. This feature has bestowed Bt as the most successful biopesticide used to date (1). During the vegetative growth phase, the bacterium is able to produce secretable proteins, including Vpb1/Vpa2 (formerly Vip1/Vip2) with activity against coleopterans and Vip3 with toxic activity against lepidopterans, whereas other proteins are produced during the stationary growth phase, including Cry and Cyt proteins (δ-endotoxins), which exhibit toxic activity against invertebrates of different orders (2). Cry proteins are mainly toxic for insect species belonging to Lepidoptera, Diptera, and Coleoptera but also against species of Nematoda (3).

**Fig 1:**
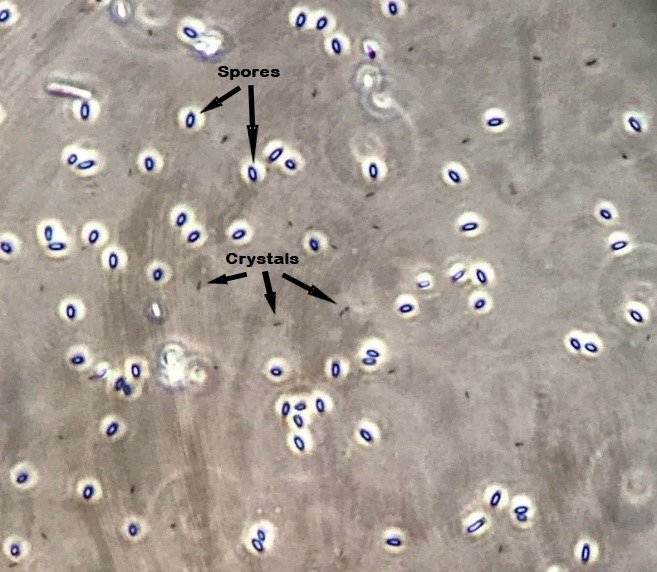
Spore and crystals from strain V-AB8.18 under phase contrast microscopy (400×).

Here, we report the draft genome sequence of a novel Bt strain designated V-AB8.18, isolated from grain storehouse dust in Albacete, Spain. The isolation was performed by suspending the sample in sterile water, heating it at 70°C for 15 min, and plating it in CCY medium (4). After incubation for 72 h at 29°C, the grown colonies were examined for the presence of crystals by phase contrast microscopy. The V-AB8.18 strain, which produced rod-shaped parasporal crystals (Fig. 1), was stored in 50% glycerol at −20°C (4).

The total DNA of the strain was purified using the Wizard Genomic DNA Purification Kit (Promega, USA) following the manufacturer’s instructions (Cat. No. A1125), after growing the strain in LB medium overnight at 29°C with shaking at 200 rpm. The DNA was sent to Novogene, who constructed a pooled Illumina library using the kit Novogene NGS DNA Library Prep Set (Cat No. PT004, Novogene, UK) and performed the sequencing using the Illumina Sequencing PE150 platform. A total of 17,722,458 raw reads with about 150 bp in size were obtained, trimmed, and assembled using Velvet v1.2.10 (5), which is included in Geneious R11 software (www.geneious.com). Gene prediction and annotation were performed with the NCBI Prokaryotic Genome Annotation Pipeline (2024 release). Genes encoding potential proteins with pesticidal activity were screened with BLAST by using a database obtained from the Bacterial Pesticidal Protein Resource Center (2). General features and summarized genome annotation data are described in Table 1. The genome sequence was also analyzed for species comparison and identification using FastANI v0.1.3 software (6). The quality of the genome (percentage of completeness and contamination) was assessed using ChekM v1.0.18 (7). Default parameters were used in all software programs.

**TABLE 1:** General features and summary statistics of the V-AB8.18 genome^*a*^.

Features	Value
Assembled genome size (bp)	6,182,614
Number of contigs	308
Largest contig (bp)	280,416
Shortest contig (bp)	1,030
Average (bp)	20,073
*N*_50_ (bp)	61,545
G + C content (%)	35
Completion (%)	98.85
Contamination (%)	0.29
Estimated coverage (×)	430
**NCBI Prokaryotic Genome Annotation**	**Value**
Genes (total)	6,532
CDSs (total)	6,479
Genes (coding)	6,151
CDSs (with protein)	6,151
Genes (RNA)	53
rRNAS	1, 1 (16S, 23S)
Complete rRNAs	1, 1 (16S, 23S)
tRNAS	46
ncRNAs	5
Pseudogenes (total)	328
CDSs (without protein)	328
Pseudogenes (frameshifted)	155 of 328
Pseudogenes (incomplete)	154 of 328
Pseudogenes (internal stop)	101 of 328
**Pesticidal proteins**	**Percentage of pairwise similarity**
App6	99
Cry5	99

^*a*^ CDS, coding sequences.

The strain V-AB8.18 showed 98.6% average nucleotide identity with *B. thuringiensis* serovar Berliner type strain ATCC 10792 (accession no. NZ_CM000753), which was consistent with the production of parasporal crystals. In addition, the screening of sequences coding for pesticidal proteins showed that the strain harbors two genes encoding potential nematicidal proteins namely, App6 (formerly Cry6) and Cry5 (Table 1). These App6 and Cry5 proteins showed 99% pairwise identity with App6Aa2 and Cry5Ba2 proteins toxic to *Meloidogyne incognita* (8) and *Meloidogyne hapla* (9), respectively, strongly suggesting the potential activity of strain V-AB8.18 against nematodes.

## Data Availability

The whole-genome shotgun project for *Bacillus thuringiensis* strain V-AB8.18 has been deposited in DDBJ/ENA/GenBank under accession number JBAKCA000000000 and the raw Illumina reads deposited in Sequence Read Archive (SRA) under the accession number SRR28083414. The assembled version described in this paper is the first version.
